# Supplementary material for: Cryo-EM structures of the BAF-Lamin A/C complex bound to nucleosomes
Source: Nat Commun. 2025 Feb 10;16:1495. doi: 10.1038/s41467-025-56823-9 (PMC11811190; doi:10.1038/s41467-025-56823-9)
Supplement: Supplementary file 1 — Supplementary Information [file 41467_2025_56823_MOESM1_ESM.pdf]

Supplementary Information for

Cryo-EM structures of the BAF-Lamin A/C complex bound to nucleosomes

Naoki Horikoshi[1,2], Ryosuke Miyake[1,3], Chizuru Sogawa-Fujiwara[1], Mitsuo Ogasawara[1], Yoshimasa Takizawa[1,4], Hitoshi Kurumizaka[1,3,5]*

[1]Laboratory of Chromatin Structure and Function, Institute for Quantitative Biosciences, The University of Tokyo, 1-1-1 Yayoi, Bunkyo-ku, Tokyo 113-0032, Japan.

[2]Department of Cell Biology and Anatomy, Graduate School of Medicine, The University of Tokyo, 1-1-1 Yayoi, Bunkyo-ku, Tokyo 113-0032, Japan.

[3]Department of Biological Sciences, Graduate School of Science, The University of Tokyo, 1-1-1 Yayoi, Bunkyo-ku, Tokyo 113-0032, Japan.

[4]Department of Computational Biology and Medical Sciences, Graduate School of Frontier Sciences, The University of Tokyo, 1-1-1 Yayoi, Bunkyo-ku, Tokyo 113-0032, Japan.

[5]Laboratory for Transcription Structural Biology, RIKEN Center for Biosystems Dynamics Research, 1-7-22 Suehiro-cho, Tsurumi-ku, Yokohama 230-0045, Japan.

*To whom correspondence should be addressed; E-mail address: kurumizaka@iqb.u-tokyo.ac.jp

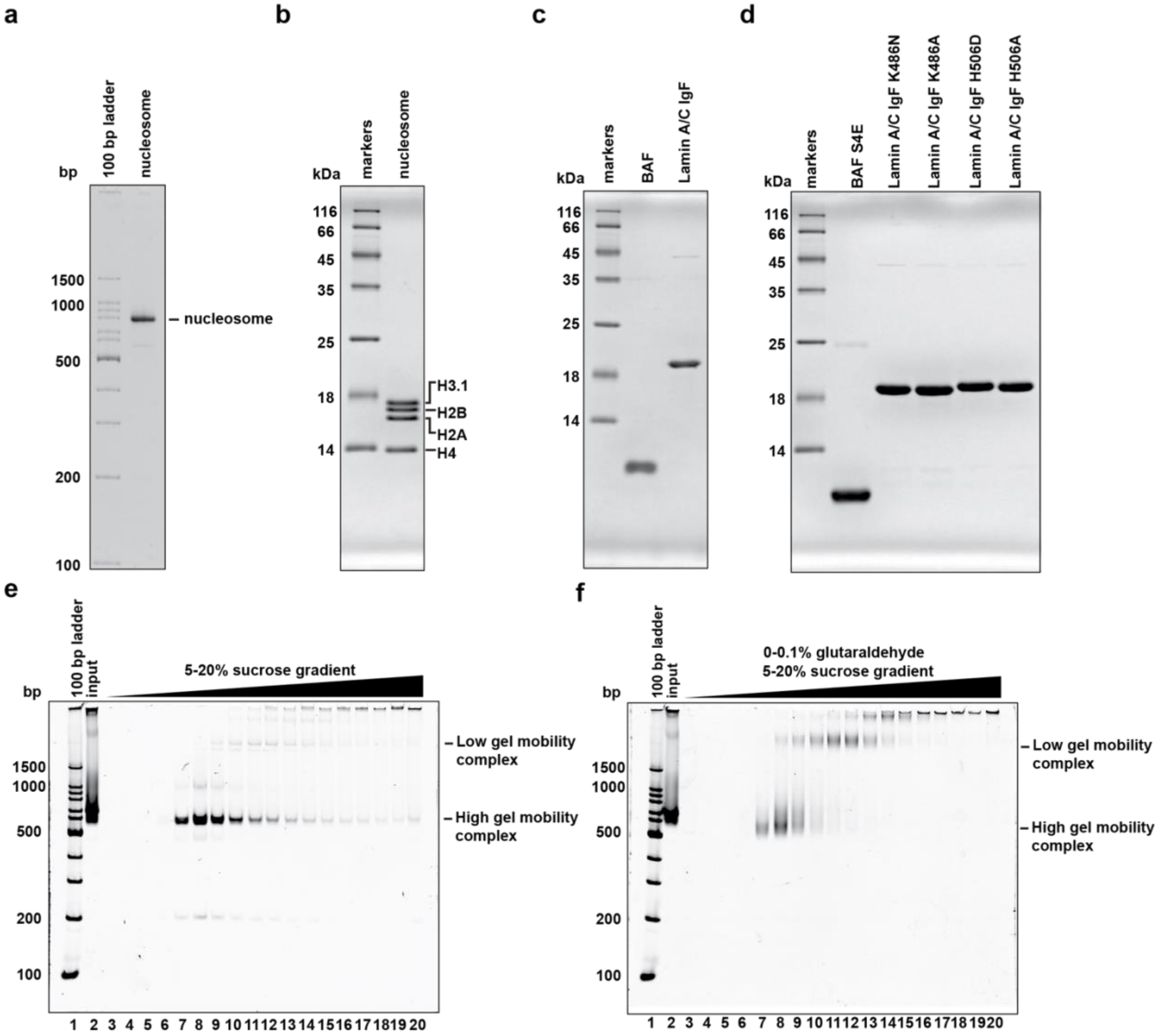

Supplementary Figure 1: Sample preparation. a,b, The nucleosome containing the Widom 193 base-pair DNA was analyzed by 6% native-polyacrylamide gel electrophoresis with ethidium bromide staining (**a**) and by 18% SDS-polyacrylamide gel electrophoresis with CBB staining (**b**). **c,** Purified BAF and Lamin A/C IgF were analyzed by 18% SDS-polyacrylamide gel electrophoresis with CBB staining. **d,** Purified BAF S4E, Lamin A/C IgF K486N, Lamin A/C IgF

K486A, Lamin A/C IgF H506D, and Lamin A/C IgF H506A were analyzed by 18% SDS-polyacrylamide gel electrophoresis with CBB staining. **e,** The BAF-Lamin A/C IgF-nucleosome complexes were fractionated by 5-20% sucrose gradient ultracentrifugation. The resulting fractions were analyzed by 5% native-polyacrylamide gel electrophoresis with SYBR Gold staining. **f,** The BAF-Lamin A/C IgF-nucleosome complexes were fractionated by 5-20% sucrose gradient ultracentrifugation in the presence of 0-0.1% glutaraldehyde. The fractionated samples were analyzed by 5% native-polyacrylamide gel electrophoresis with SYBR Gold staining.

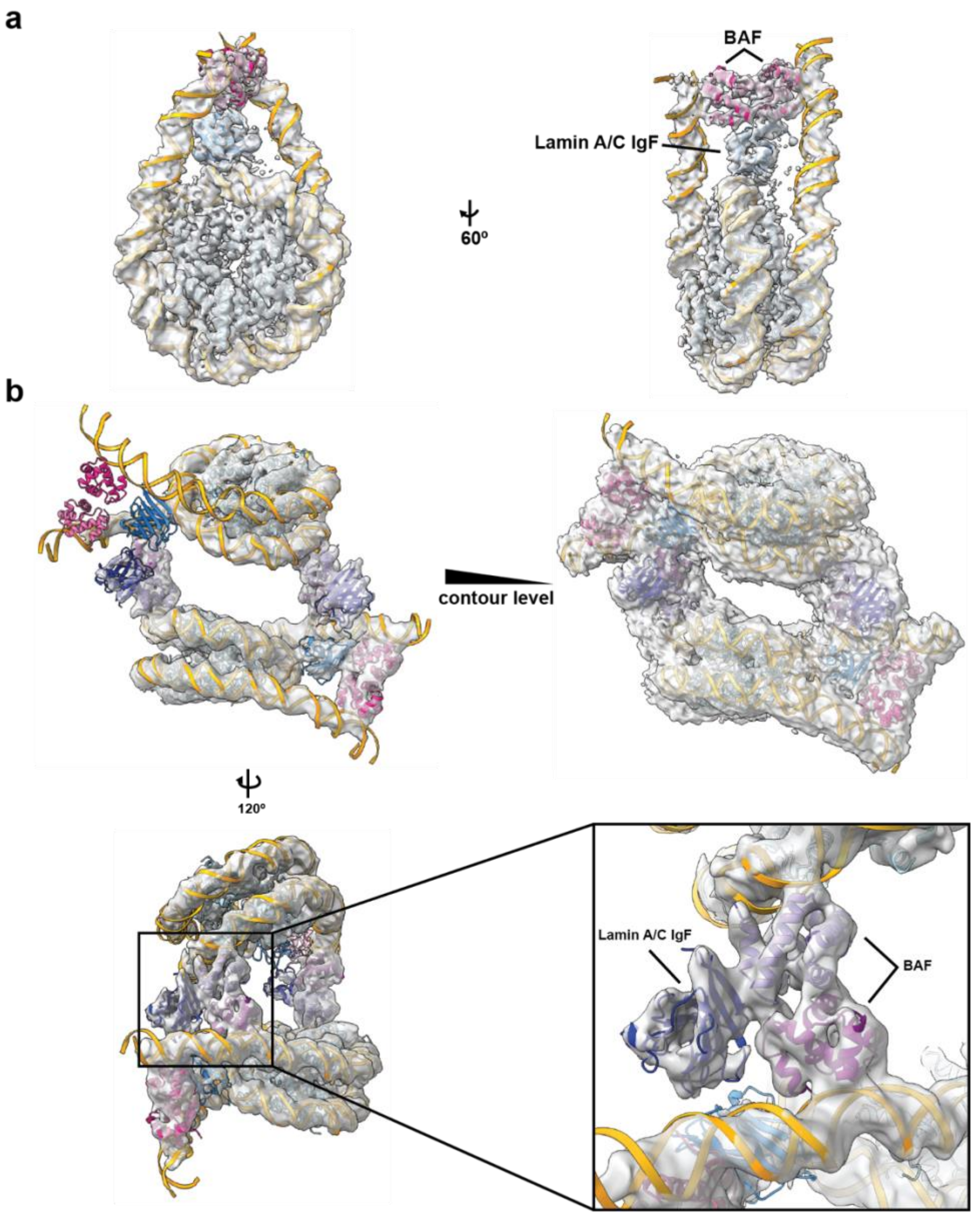

Supplementary Figure 2: Structures of the BAF-Lamin A/C IgF-nucleosome complexes. a, Model and density map of the BAF-Lamin A/C IgF-nucleosome complex corresponding to the high gel mobility complex are overlaid. **b,** Model and density map of the BAF-Lamin A/C IgF-nucleosome complex corresponding to the low gel mobility complex are overlaid. Two density maps with different contour levels are presented. The black box in the bottom-left panel encloses the region of the Lamin A/C IgF and BAF dimer bridging the nucleosomes, shown in a close-up view in the right panel.

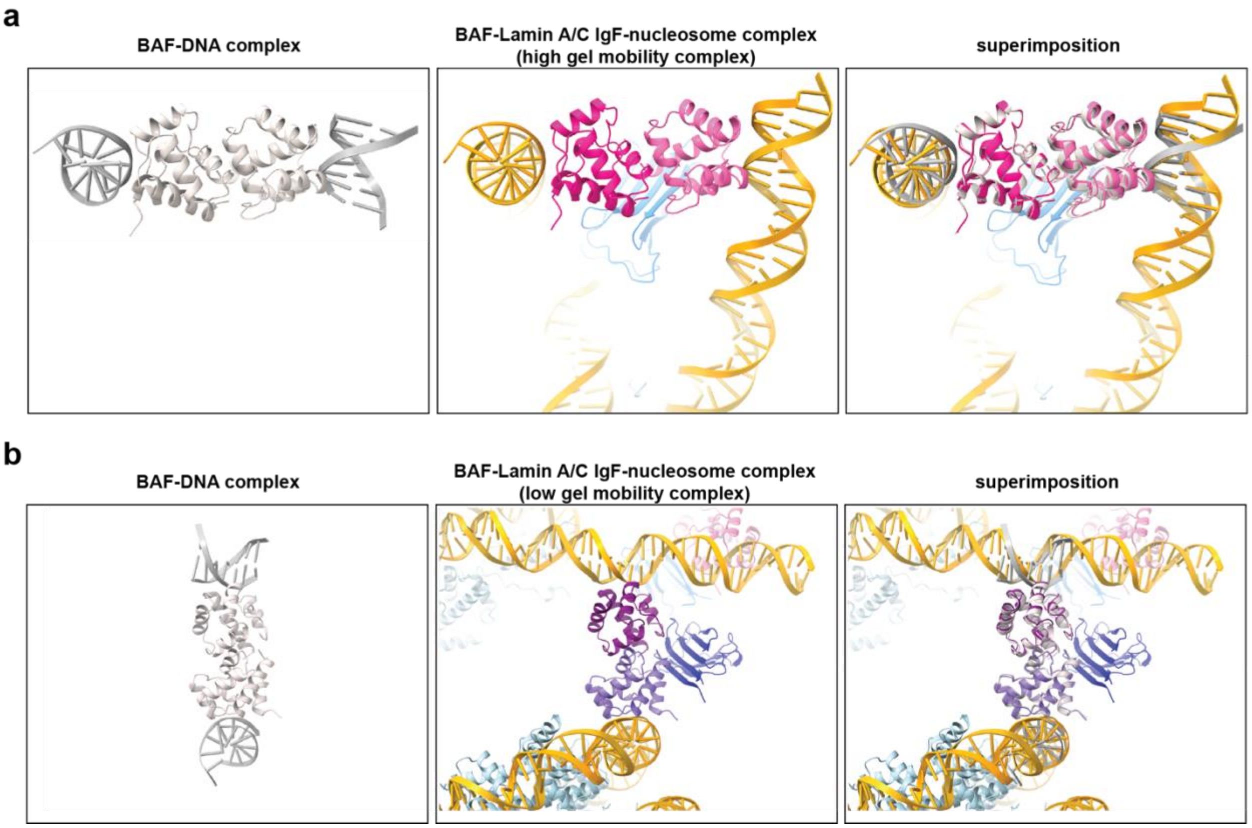

Supplementary Figure 3: Orientation of DNAs bound to the BAF dimer in the complexes. a, Comparison of the orientations of DNAs bound to the BAF dimer in the BAF-DNA complex (left panel), the BAF-Lamin A/C IgF-nucleosome complex corresponding to the high gel mobility complex (center panel), and their superimposition (right panel). **b,** Comparison of the orientations of DNAs bound to the BAF dimer in the BAF-DNA complex (left panel), the BAF-Lamin A/C IgF-nucleosome complex corresponding to the low gel mobility complex (center panel), and their superimposition (right panel).

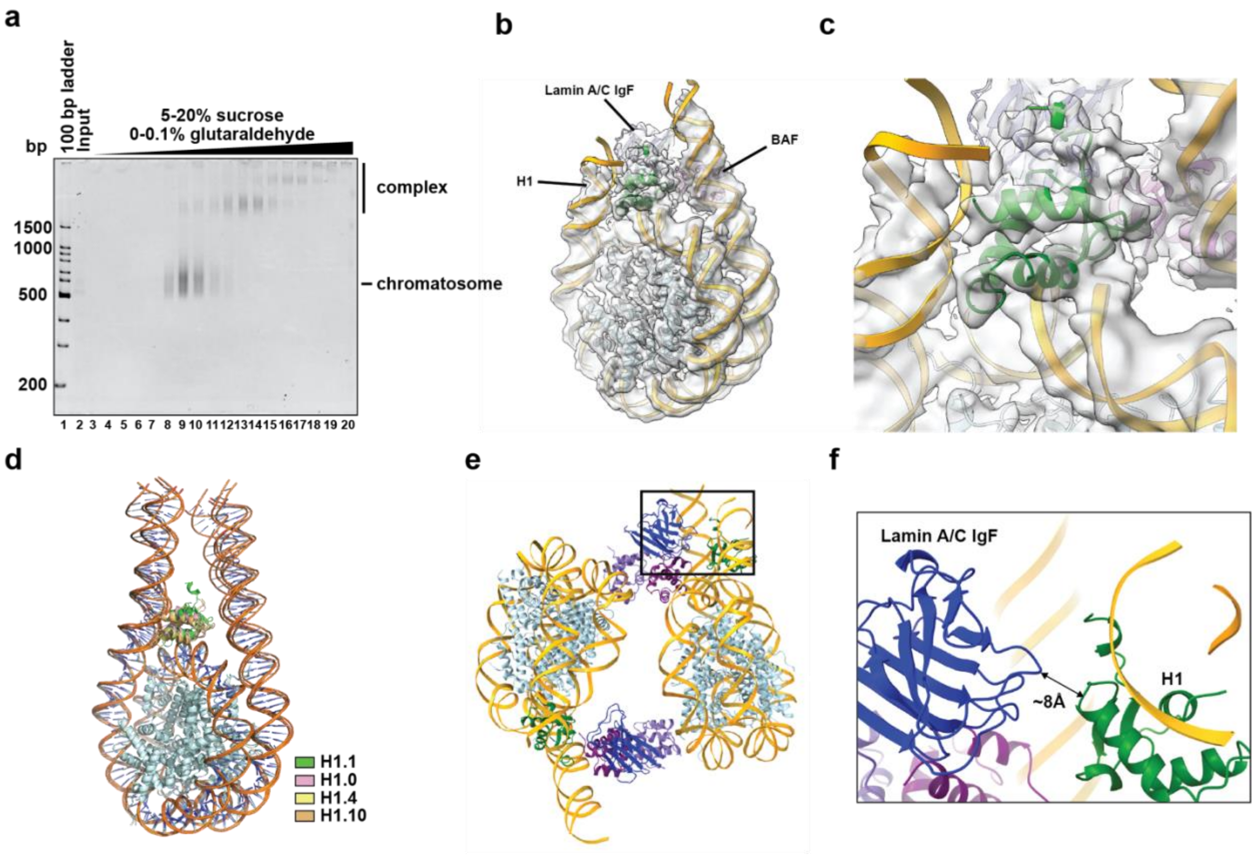

Supplementary Figure 4: Structure of the BAF-Lamin A/C IgF-H1-nucleosome complex. a, The BAF-Lamin A/C IgF-H1-nucleosome complex was fractionated by 5-20% sucrose gradient ultracentrifugation with 0-0.1% glutaraldehyde. The fractionated samples were analyzed by 5% native-polyacrylamide gel electrophoresis with ethidium bromide staining. Fractions corresponding to lanes 11-15 were collected and used for cryo-EM analysis. **b,** Cryo-EM map of the BAF-Lamin A/C IgF-H1-nucleosome complex. **c,** Close-up view of H1 in the structure of the BAF-Lamin A/C IgF-H1-nucleosome complex. **d**, Structural comparison of the chromatosome in the BAF-Lamin A/C IgF-H1-nucleosome complex with chromatosomes containing H1 subtypes H1.0 (PDB ID: 7K5X), H1.4 (PDB ID: 7K5Y), and H1.10 (PDB ID: 7K60). **e**, Overall structure of the BAF-Lamin A/C IgF-H1-nucleosome complex. **f**, Close-up view of the black-boxed region in panel e, highlighting the area around Lamin A/C IgF and the globular domain of H1 in the BAF-Lamin A/C IgF-H1-nucleosome complex.

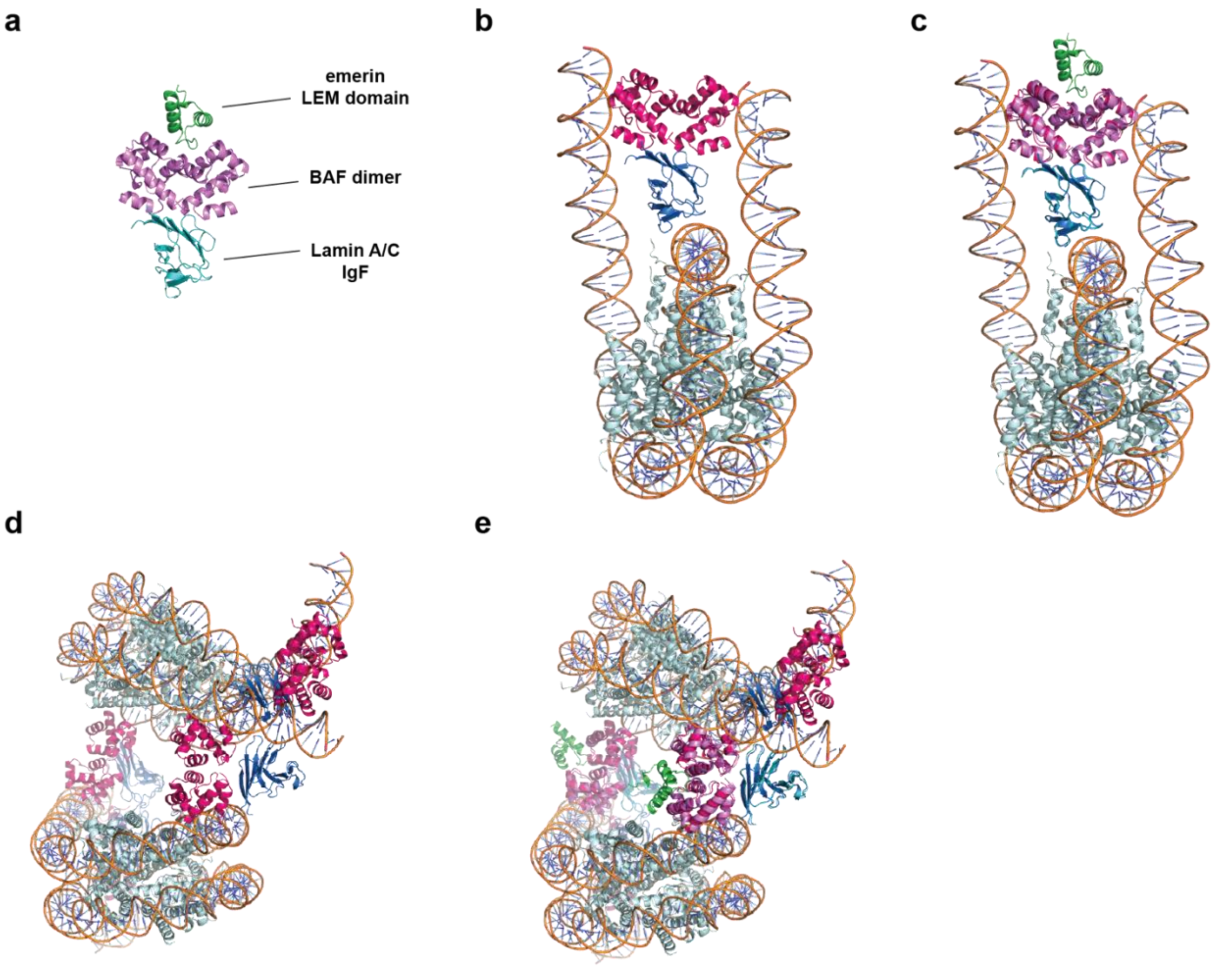

Supplementary Figure 5: Potential binding of emerin in the BAF-Lamin A/C IgF-nucleosome complex. a, The previously published structure of the BAF-Lamin A/C IgF-emerin LEM domain (PDB ID: 6GHD). **b,** Structure of the BAF-Lamin A/C-nucleosome complex corresponding to the high gel mobility complex. The BAF dimer, Lamin A/C IgF, and histone complex are colored pink, blue, and pale cyan, respectively. **c,** Superimposed view of panels a and b, showing the potential positioning of emerin in the high gel mobility complex. **d,** Structure of the BAF-Lamin A/C-nucleosome complex corresponding to the low gel mobility complex. **e,** Superimposed view of panels a and d, showing the potential positioning of emerin in the low gel mobility complex.

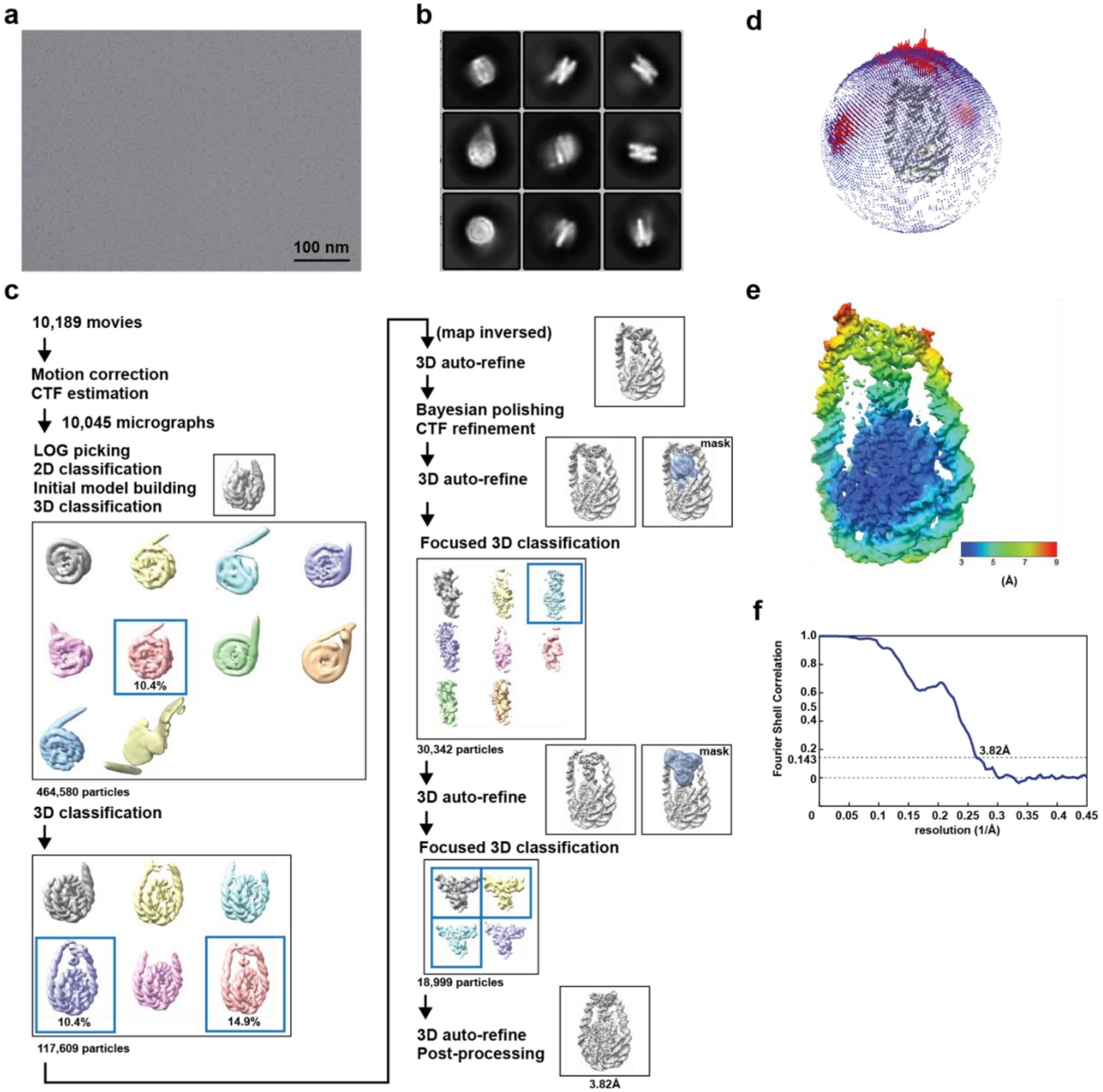

Supplementary Figure 6: Workflow of the image processing of the cryo-EM dataset for Fraction 1 of the BAF-Lamin A/C IgF-nucleosome complex. a, Representative micrograph of Fraction 1 of the BAF-Lamin A/C IgF-nucleosome complex. 10,189 micrographs were collected. **b,** Representative 2D class averages of the complex. **c,** Flow chart of data processing of the complex. **d,** Euler angle distribution of the 3D reconstruction of the complex. **e,** Local resolution map of the complex. **f,** The gold standard Fourier Shell Correlation (FSC) curve of the complex, calculated from 3D reconstructions generated by two halves of datasets.

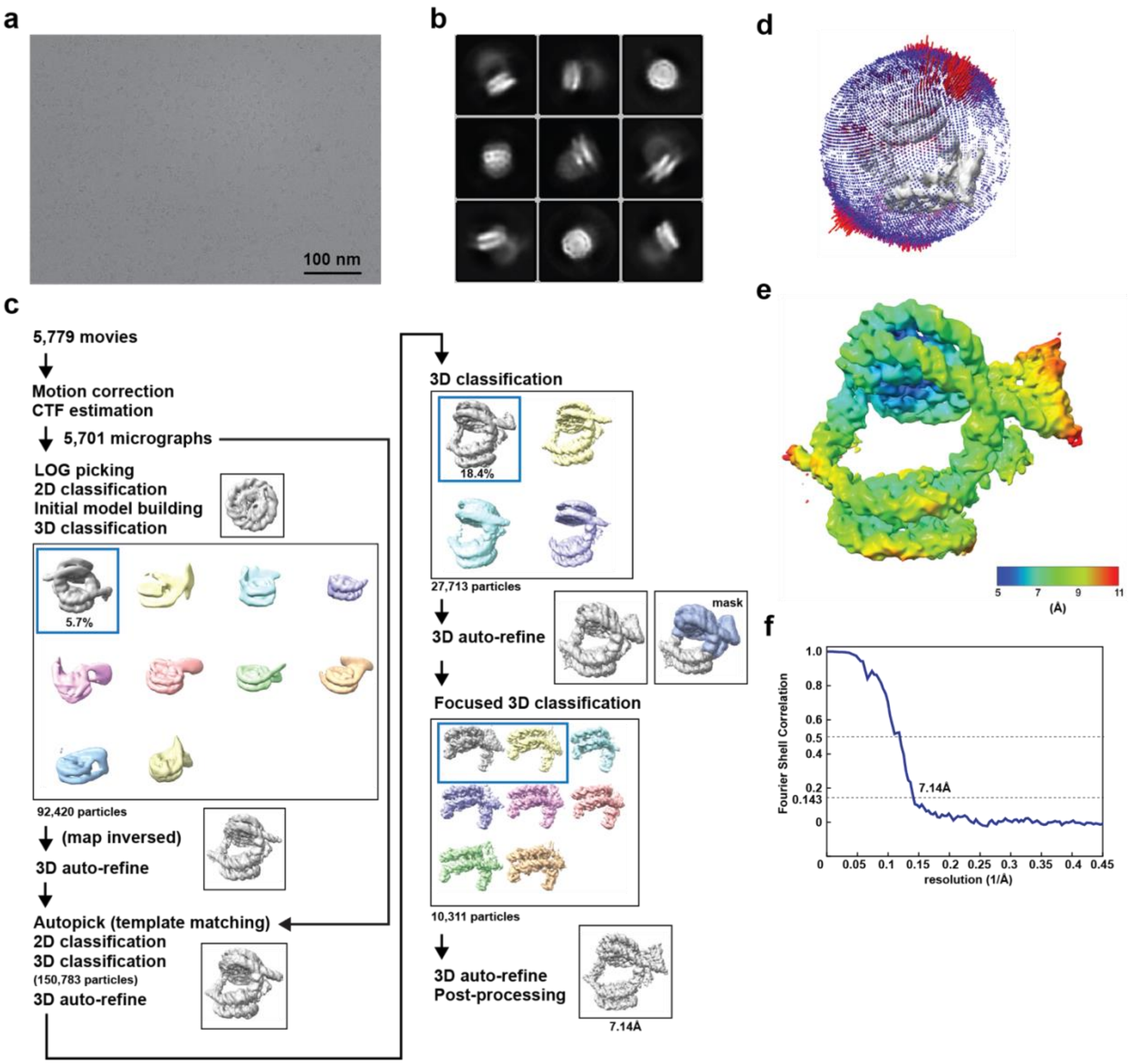

Supplementary Figure 7: Workflow of the image processing of the cryo-EM dataset for Fraction 2 of the BAF-Lamin A/C IgF-nucleosome complex. a, Representative micrograph of Fraction 2 of the BAF-Lamin A/C IgF-nucleosome complex. 5,779 micrographs were collected. **b,** Representative 2D class averages of the complex. **c,** Flow chart of data processing of the complex. **d,** Euler angle distribution of the 3D reconstruction of the complex. **e,** Local resolution map of the complex. **f,** The gold standard Fourier Shell Correlation (FSC) curve of the complex, calculated from 3D reconstructions generated by two halves of datasets.

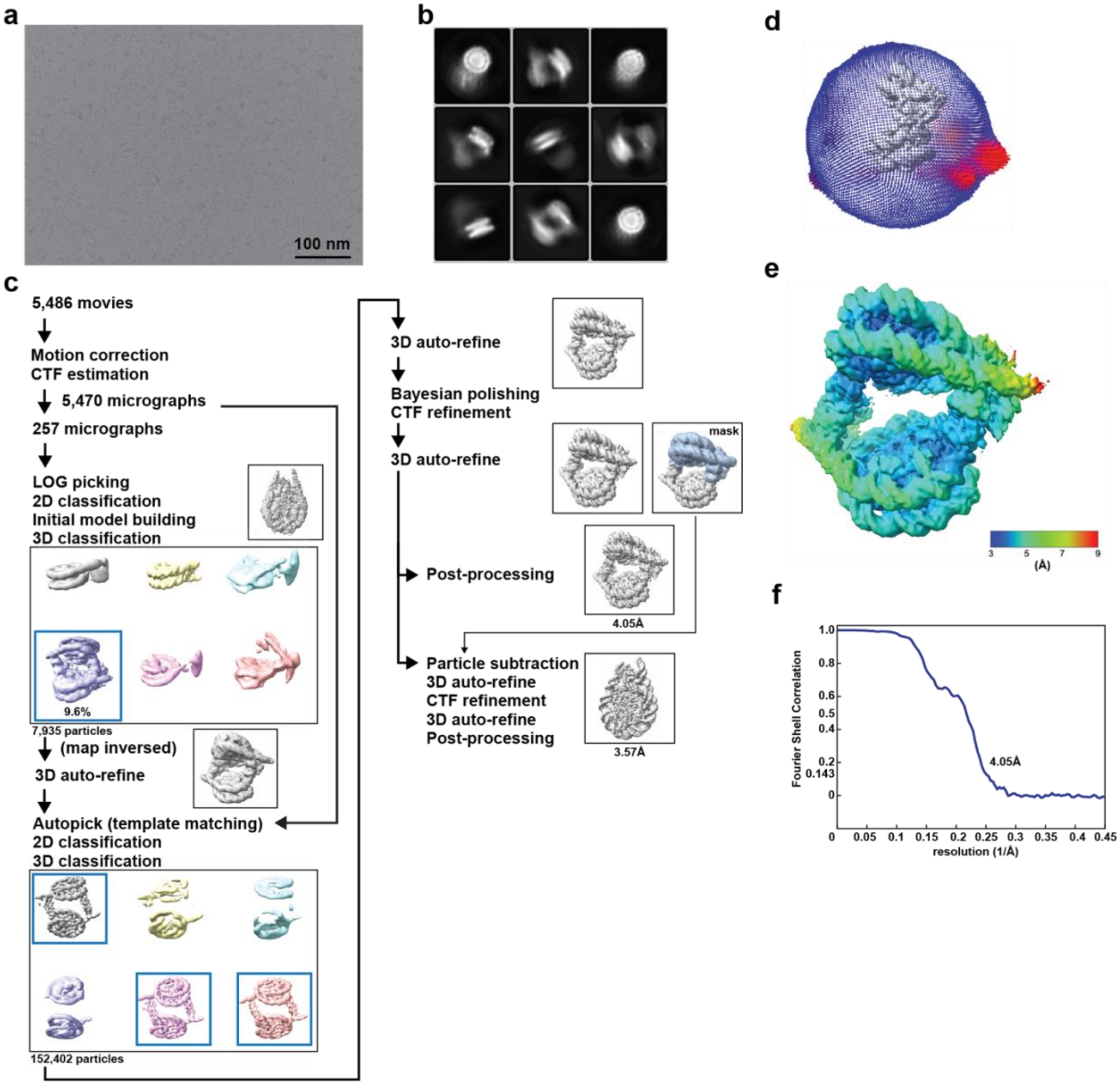

Supplementary Figure 8: Workflow of the image processing of the cryo-EM dataset for the BAF-Lamin A/C IgF-H1.1-nucleosome complex. a, Representative micrograph of the BAF-Lamin A/C IgF-H1-nucleosome complex. 5,486 micrographs were collected. **b,** Representative 2D class averages of the complex. **c,** Flow chart of data processing of the complex. **d,** Euler angle distribution of the 3D reconstruction of the complex. **e,** Local resolution map of the complex. **f,** The gold standard Fourier Shell Correlation (FSC) curve of the complex, calculated from 3D reconstructions generated by two halves of datasets.

Supplementary Table 1: Cryo-EM data collection, refinement and validation statistics.

	#1 BAF-Lamin A/C IgF bound to the nucleosome (High mobility complex) (EMD-61231) (PDB 9J8M)	#2 BAF-Lamin A/C IgF bound to the nucleosome (Low mobility complex) (EMD-61232) (PDB 9J8N)	#3 BAF-Lamin A/C IgF bound to the chromatosome containing H1.1 (EMD-61233) (PDB 9J8O)
**Data collection and processing**			
Magnification	81,000×	81,000×	81,000×
Voltage (kV)	300	300	300
Electron exposure ($e^{-}/Å^2$)	60.4	60.1	59.5
Defocus range (μm)	-1.0 to -2.5	-1.0 to -2.5	-1.0 to -2.5
Pixel size (Å)	1.06	1.06	1.06
Symmetry imposed	C1	C1	C1
Initial particle images (no.)	5,720,577	2,697,637	3,105,378
Final particle images (no.)	18,999	10,311	152,402
Map resolution (Å)	3.82	7.14	4.05
FSC threshold	0.143	0.143	0.143
Map resolution range (Å)	3.36 to 10	5.14 to 17.8	3.58 to 13.7
**Refinement**			
Initial model used (PDB code)	5B0Z, 7K5X, and 6GHD	5B0Z, 7K5X, and 6GHD	5B0Z, 7K5X, 6GHD, and AF-Q02539-F1-model_v4 (AlphaFold2)
Model resolution (Å)	4.05	8.03	4.48
FSC threshold	0.5	0.5	0.5
Map sharpening *B* factor ($Å^2$)	13.8	0.7	-72.361
Model composition			
Non-hydrogen atoms	15,831	36,716	32,116
Protein residues	1,047	2,684	2,250
Nucleotide	368	756	704
B factors ($Å^2$)			
Protein	35.92	38.63	39.30
Nucleotide	101.07	104.69	96.77
R.m.s. deviations			
Bond lengths (Å)	0.003	0.005	0.007
Bond angles (°)	0.521	0.611	0.670
Validation			
MolProbity score	1.43	2.03	1.75
Clashscore	5.47	17.92	3.92
Poor rotamers (%)	1.49	2.28	3.38
Ramachandran plot			
Favored (%)	99.12	98.44	97.05
Allowed (%)	0.88	1.56	2.95
Disallowed (%)	0	0	0
